# Investigating associations between long-term poverty exposure and premature mortality: evidence from the National Longitudinal Survey of Youth 1979 prospective cohort

**DOI:** 10.1016/S2468-2667(25)00227-0

**Published:** 2025-11

**Authors:** Calvin L Colvin, Samuel L Swift, Xuexin Yu, Katrina L Kezios, Adina Zeki Al Hazzouri

**Affiliations:** Department of Epidemiology, Mailman School of Public Health, Columbia University, New York, NY, USA; College of Population Health, University of New Mexico Health Sciences Center, Albuquerque, NM, USA; Department of Epidemiology, Mailman School of Public Health, Columbia University, New York, NY, USA; Department of Epidemiology, Mailman School of Public Health, Columbia University, New York, NY, USA, Department of Epidemiology, Boston University School of Public Health, Boston, MA, USA; Department of Epidemiology, Mailman School of Public Health, Columbia University, New York, NY, USA

## Abstract

**Background:**

Living in poverty increases the risk for mortality. Existing research that examines life course poverty typically relies on measures separated by decades of time. Here, we aimed to estimate the association of 20-year cumulative poverty exposure from emerging adulthood through to established adulthood with premature mortality assessed over the following 15 years.

**Methods:**

We included National Longitudinal Survey of Youth 1979 study participants with three or more family income measures between 1985 and 2004. Participants were, on average, aged 23 years at the start and aged 42 years at the end of this period. Follow-up for premature mortality began in 2004 and ended in 2019, at which time participants were aged 53–62 years. We defined cumulative poverty by the proportion of family size-adjusted income measures less than 200% of the Federal Poverty Level: never in poverty, sometimes in poverty (>0 and less than a third of measures), often in poverty (a third or more but not all measures), and always in poverty. Primary analyses used confounder-adjusted Cox proportional hazards regression models. Our outcome was mortality between 2004 and 2019.

**Findings:**

Our sample included 5653 participants, with 1484 (26·2%) never in poverty, 1867 (33·0%) sometimes in poverty, 1852 (32·8%) often in poverty, and 450 (8·0%) always in poverty. 363 (6·4%) participants were reported deceased over follow-up. Compared with participants never in poverty, those sometimes, often, and always in poverty had 1·10 (95% CI 0·79–1·53), 1·53 (1·09–2·14), and 2·53 (1·61–3·96) times higher rates of premature mortality, respectively.

**Interpretation:**

Greater cumulative exposure to poverty across emerging and established adulthood is associated with a greater risk for premature mortality. To inform public health action and policy, future research should evaluate the effects of providing support to individuals who are experiencing financial hardships during these important life stages on health and longevity.

**Funding:**

National Institute of Health’s National Institute on Aging.

## Introduction

Millions of individuals in the USA experience poverty.^1^ Income in general and poverty in particular are important social determinants of health, contributing to an individuals’ health behaviours,^2^ their access to health-preserving goods and services such as health care,^3^ and their exposure to harmful environmental conditions.^4^ Many studies show that low incomes and poverty are associated with increased mortality risk and lower life expectancy.^5–8^ However, previous studies often relied on income measures collected at a single point in time^6–8^ or at infrequent points across the life course.^9^

There are several reasons why poverty would be examined as a cumulative life course exposure rather than cross-sectionally or with a few timepoints. Studies of income and financial wellbeing measured repeatedly over time suggest that longer durations of time in poverty are associated with poorer health outcomes in a dose-dependent manner.^5,10,11^ By only considering income at one timepoint or a diffuse set of timepoints, the nuances in exposure might be missed. Additionally, income is not static over the life course. Although, on average, an individual’s income increases from emerging adulthood (ie, those aged 18–29 years) through to established adulthood (ie, those aged 30–45 years) before peaking in midlife,^12^ there is heterogeneity in income dynamics,^13^ and many US adults have a bout of poverty or cycle in and out of poverty.^14,15^ Such heterogeneity is missed when a few datapoints are used to summarise income exposure over many years. Furthermore, a growing volume of literature suggests that economic disadvantage during emerging and established adulthood can substantially affect long-term health and increase the risk of premature mortality.^16,17^ Yet, to the best of our knowledge, no previous work has examined whether a cumulative amount of exposure to low income from emerging through to established adulthood is associated with premature mortality.

In this study, we aimed to examine the relationship between cumulative poverty and premature mortality using data spanning 30 years from the National Longitudinal Survey of Youth 1979 (NLSY79), a national prospective cohort study of adults in the USA.

## Methods

### Study population and analytical sample

The NLSY79 is an ongoing national longitudinal cohort study of US-residing men and women who were born between 1957 and 1964 and were aged between 14 years and 22 years at the time of their first interview in 1979.^18^ The NLSY79 interviewed participants every year between 1979 and 1994 and biennially thereafter. The original NLSY79 cohort was composed of multiple sub-samples.

We restricted our analytic sample to NLSY79 participants who were not part of a discontinued sub-sample. We further restricted the sample to participants who completed an interview and had a non-missing family income measure at the beginning of our exposure period in 1985 and who had, at the minimum, two additional non-missing family income measures through to the end of the exposure period in 2004. Participants could contribute up to 15 total family income measures during the exposure period. To ensure that all participants were alive and remained as part of the study at the start of mortality follow-up, we required that participants completed an interview at the end of the exposure period in 2004. Finally, we required complete data on the covariates, for a complete case analytic sample size of 5653. We used data that were de-identified and publicly available and so this analysis did not require Institutional Review Board approval.

### Exposure: cumulative poverty from 1985 to 2004

For each interview wave, participants were asked to report their pre-tax income from several sources (ie, employment, businesses owned, unemployment compensation, child support, alimony, government welfare programmes, employment-related pensions, individual retirement accounts, retirement annuities, government social security programmes, military income, veterans benefits and disability payments, workers compensation programmes, estates, trusts, inheritances, gifts, family financial transfers, education grants, rental subsidies, and investment income) and the pre-tax incomes of all related household members for the year before the interview. These reported incomes were combined by the NLSY79 into a net family income variable. We determined if participants were in poverty each interview wave using participants’ net family income, family size, and the Federal Poverty Level for their reported family size. Consistent with a seminal study that examined the relationship between poverty and health outcomes,^5^ we defined poverty as a net family income <200% of the Federal Poverty Level. We calculated the proportion of interviews in which participants’ reported family incomes that met our definition of poverty and, consistent with previous work, categorised participants into four groups: no interviews with poverty-level family income (ie, never in poverty), more than zero but fewer than a third of interviews with poverty-level family income (ie, sometimes in poverty), a third or more but fewer than all interviews with poverty-level family income (ie, often in poverty), and all interviews with poverty-level family income (ie, always in poverty).^10^

### Outcome: premature mortality between 2004 and 2019

Our outcome was mortality between 2004 and 2019. We considered mortality in this period to be premature because participants would have been aged 53–62 years by the end of outcome follow-up, which is below the life expectancy for all birth years (1957–64) included in the sample.^19^ Each interview wave, the NLSY79 recorded a reason for the participants who did not complete an interview. If a participant’s reason was recorded as being deceased, we considered them to have died.

### Covariates

We examined participants’ baseline sociodemographic characteristics, health behaviours, and health status as potential confounders (measured in 1985, unless otherwise noted). Sociodemographic characteristics included age, interviewer-perceived sex, interviewer-perceived race and self-reported ethnicity, early-life cognitive ability as measured by the percentile score on the Armed Forces Qualification Test (measured in 1981), years of education, years of education completed by their most educated parent (measured in 1979), occupation, and living in the south of the USA when aged 14 years. Health behaviours included past month smoking status (measured in 1984) and past week heavy alcohol consumption (measured in 1984). We used BMI as a measure of baseline health status. All models in the main analysis also included adjustment for the number of income measures contributed across the exposure period. We provide further information on definitions for sex, race and ethnicity, Armed Forces Qualification Test scores, occupation, region of residence, BMI, and heavy alcohol consumption in the appendix (p 2).

### Statistical analysis

We summarised participants’ covariates overall and by cumulative poverty category using numbers and percentages for categorical variables and means and SDs for numeric variables. To describe the survival experience of the cohort, we calculated crude incidence rates per 1000 person-years for premature mortality overall and by cumulative poverty category and calculated crude incidence rate ratios for each cumulative poverty category relative to those never in poverty. Additionally, we reported crude excess mortality as the difference in cumulative incidence comparing those sometimes, often, and always in poverty with those never in poverty, multiplied by 100.

We used Cox proportional hazards regression to estimate the confounder-adjusted association of cumulative poverty with premature mortality. Participants contributed person-years at risk from the 15th day of their interview month in 2004 through to their date of death or the date of their last non-missing study interview before the conclusion of the 2018 NLSY79 survey wave in November, 2019. Since we lacked information on the precise date of death for participants who died, we selected the year of death as the interview wave the participant was reported as deceased and imputed the month of death to be the month of their last completed interview and the date of death to be the 15th day of the month. We tested the proportional hazards assumption using the cox.zph() function in R (version 4.5.0), which uses Schoenfeld residuals to assess proportionality. Three Cox proportional hazards models with progressive adjustment for covariates were used to calculate hazard ratios (HRs) and 95% CIs. In the first model, we adjusted only for the number of income measures contributed over the exposure period. The second model added adjustment for sociodemographic characteristics. In the third model, we added adjustment for health behaviours and health status. To visualise our results, we used the adjusted Curves package in R and the direct adjustment method to plot adjusted survival probability curves over follow-up for each cumulative poverty group. Survival probabilities were obtained from the fully adjusted Cox proportional hazards model described earlier.

### Sensitivity analyses

To examine the robustness of our main findings, we completed several post-hoc analyses. First, to test how sensitive results were to attempts at addressing bias attributable to our exclusion of participants with missing data as well as to bias attributable to participants’ incomplete record of family income measures over the exposure period, we used multiple imputation with chained equations to recover missing covariate data and all family incomes during the exposure period.^20^ The imputation was conducted among participants with at least a baseline income measure and who were known to be alive in 2004 at the start of the outcome follow-up. The missing covariate data is summarised in the appendix (p 5) as are characteristics for those included in the main analysis and those excluded due to having missing data or an insufficient number of family income measures (p 6). We imputed 10 datasets. The imputation models included all sociodemographic characteristics, health behaviours, and health status variables listed previously, the outcome, all available family income and family size measures from before and during the exposure period, occupations held during the exposure period, regions of residence during the exposure period, and marital statuses during the exposure period. We used Rubin’s Rules to pool estimates across the imputed datasets.

Next, we repeated the main analysis using 175% and 150% of the Federal Poverty Level as thresholds to define poverty. We also restricted our sample to participants with 10 or more family income measures during the exposure period and repeated the analysis among this subset. In this analysis, the proportional hazards assumption was violated for those living in the south of the USA at age 14 years. To address this, we stratified the baseline hazards by this variable. Next, we treated participants’ proportion of poverty-level family incomes as a continuous rather than categorical variable and examined the relationship between poverty and premature mortality using restricted cubic splines with four knots. Finally, participants’ values for some covariates likely changed over the exposure period because of their previous poverty while also influencing their later risk of poverty. To gauge the robustness of our findings to potential time-varying confounding in a simple analysis, we adjusted for values of these covariates at or near the end of the exposure period; if findings hold, this suggests that observed associations are unlikely due to time-varying confounding. R version 4.5.0 was used for analyses.

### Role of the funding source

The funder of the study had no role in study design, data collection, data analysis, data interpretation, or writing of the report.

## Results

**Figure 1:**
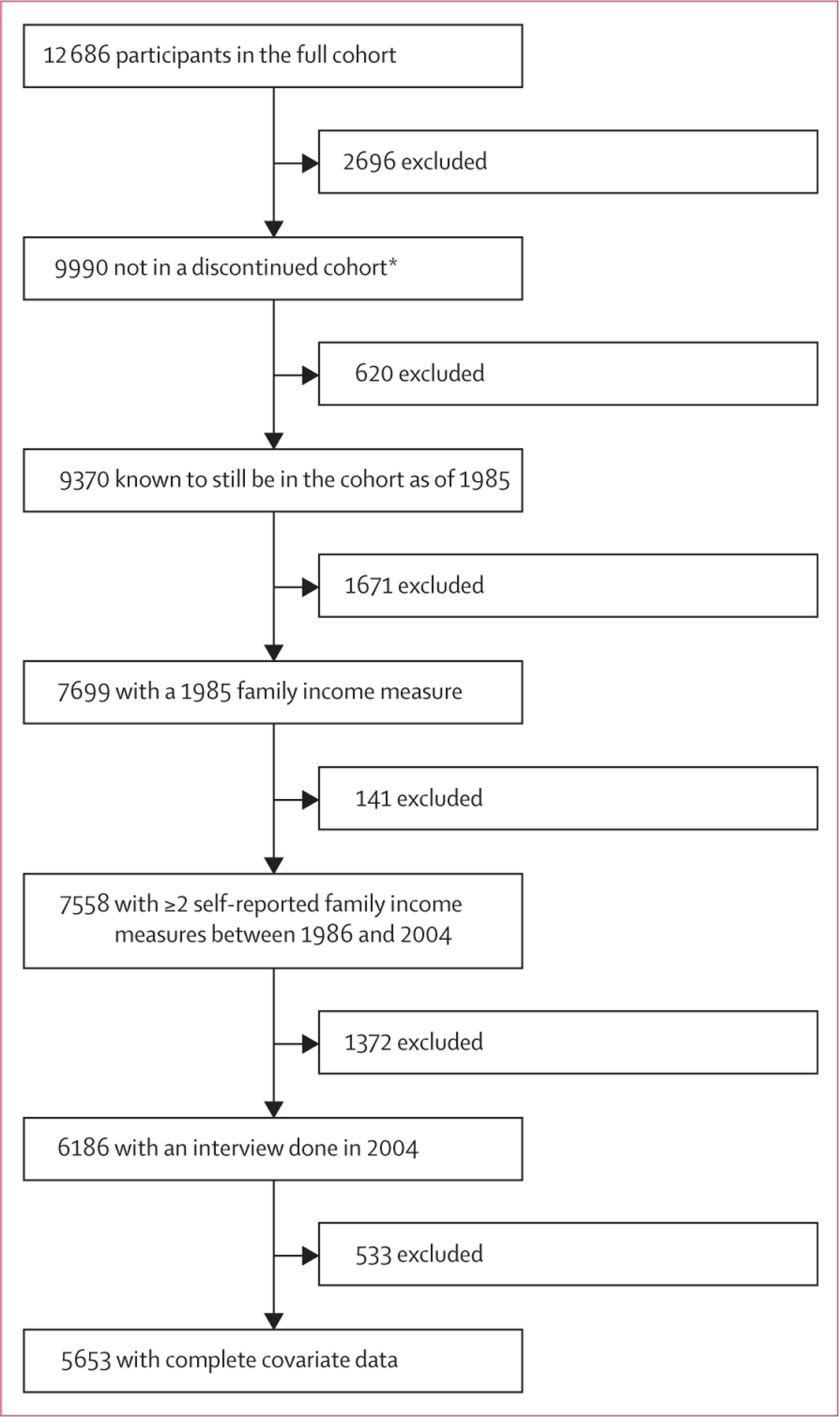
Analytical sample flowchart of the National Longitudinal Survey of Youth, 1979 *The cohort initially included an oversample of youth in the military and economically disadvantaged Black, Hispanic, and non-Black and non-Hispanic youth. The military oversample was discontinued in 1984. The oversample of economically disadvantaged non-Black and non-Hispanic youth was discontinued in 1990.

The original NLSY79 cohort was composed of 12 686 participants in all (figure 1). After exclusions, 5653 participants had complete covariate data and were included in the analysis. On average, participants were aged 23·5 years in 1985, the beginning of the exposure period (table 1). 1484 (26·3%) of 5653 participants were never in poverty, 1867 (33·0%) sometimes in poverty, 1852 (32·8%) often in poverty, and 450 (8·0%) always in poverty. The proportion of participants that were female, Black, Hispanic, who lived in the south of the USA at age 14 years, who currently smoked, and who were unemployed increased as cumulative exposure to poverty increased, whereas mean years of participants’ education, parents’ years of education, and the proportion who had heavy alcohol consumption decreased as cumulative poverty exposure increased. In addition, mean BMI increased as cumulative poverty exposure increased and Armed Forces Qualification Test percentile score and number of income measures decreased. 5024 (88·9%) eligible participants contributed at least 10 family income measurements.

**Figure 2:**
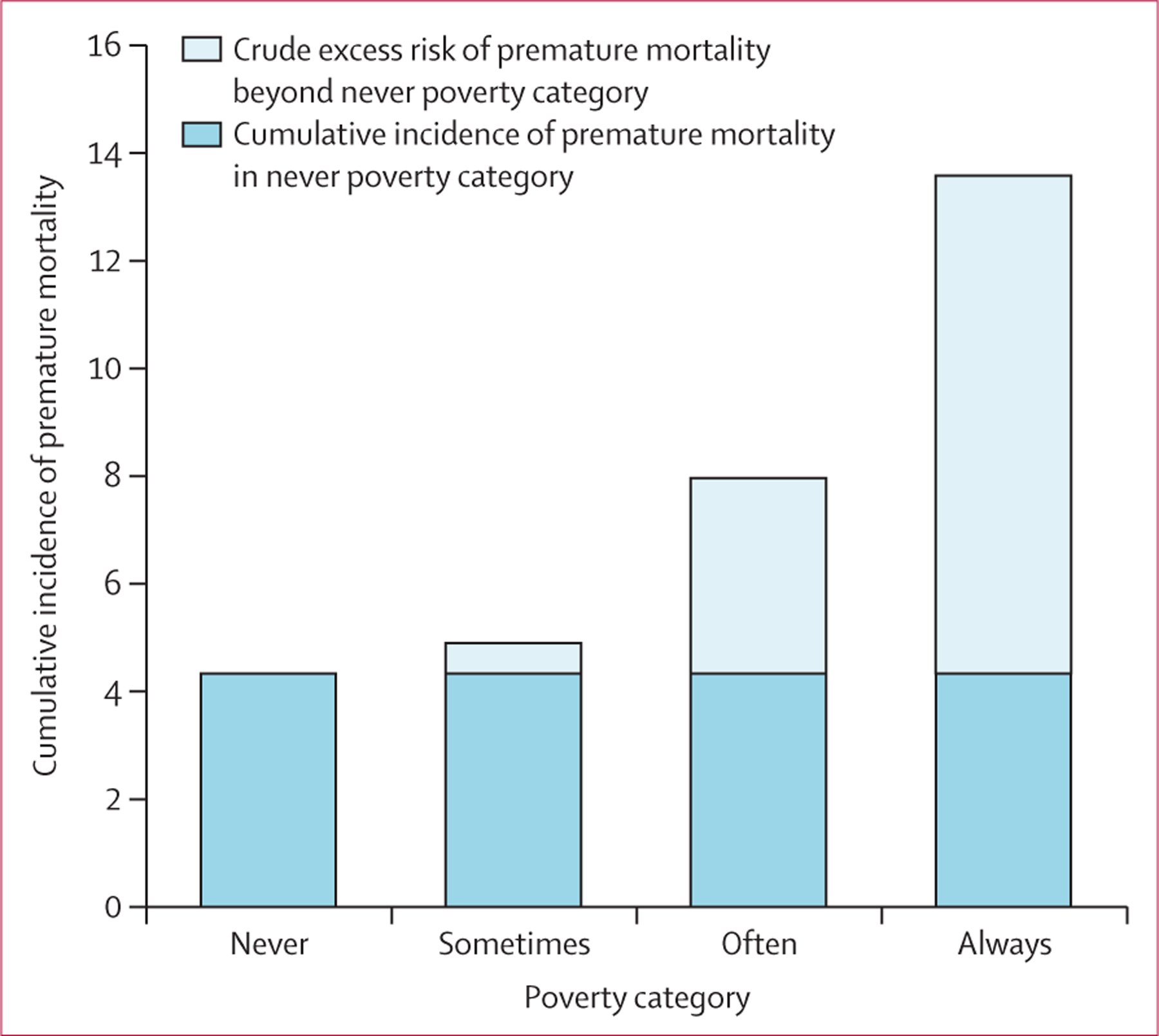
Cumulative incidence and crude excess risk of premature mortality (2004–18) associated with cumulative poverty from emerging adulthood to established adulthood (1985–2004), National Longitudinal Survey of Youth, 1979 The full length of each bar represents cumulative incidence. Excess risk (relative to risk in the never poverty group shown by the dark blue part of the bar) is indicated by the light blue part of the bar. The never group had no interviews in poverty (n=1484), the sometimes group had more than zero but fewer than a third of interviews in poverty (n=1867), the often group had more than a third but fewer than all interviews in poverty (n=1852), and the always group had all interviews in poverty (n=450).

363 (6·4%) deaths were reported between 2004 and 2019, and the median follow-up was 14·5 person-years (IQR 14·3–14·7). The overall crude incidence rate for premature mortality was 4·75 per 1000 person-years (95% CI 4·26–5·24). Incidence rates for those never, sometimes, often, and always in poverty were 3·19 (2·41–3·97), 3·60 (2·86–4·34), 5·88 (4·93–6·83), and 10·06 (7·54–12·59) per 1000 person-years, respectively, and incident rate ratios for those sometimes, often, and always in poverty compared with never in poverty were 1·13 (0·82–1·56), 1·84 (1·38–2·49), and 3·15 (2·22–4·48), respectively (appendix p 7). In addition, the crude excess risk for premature mortality (compared with participants never in poverty) was 0·56% for participants sometimes in poverty, 3·62% for those often in poverty, and 9·24% for those always in poverty (figure 2).

**Figure 3:**
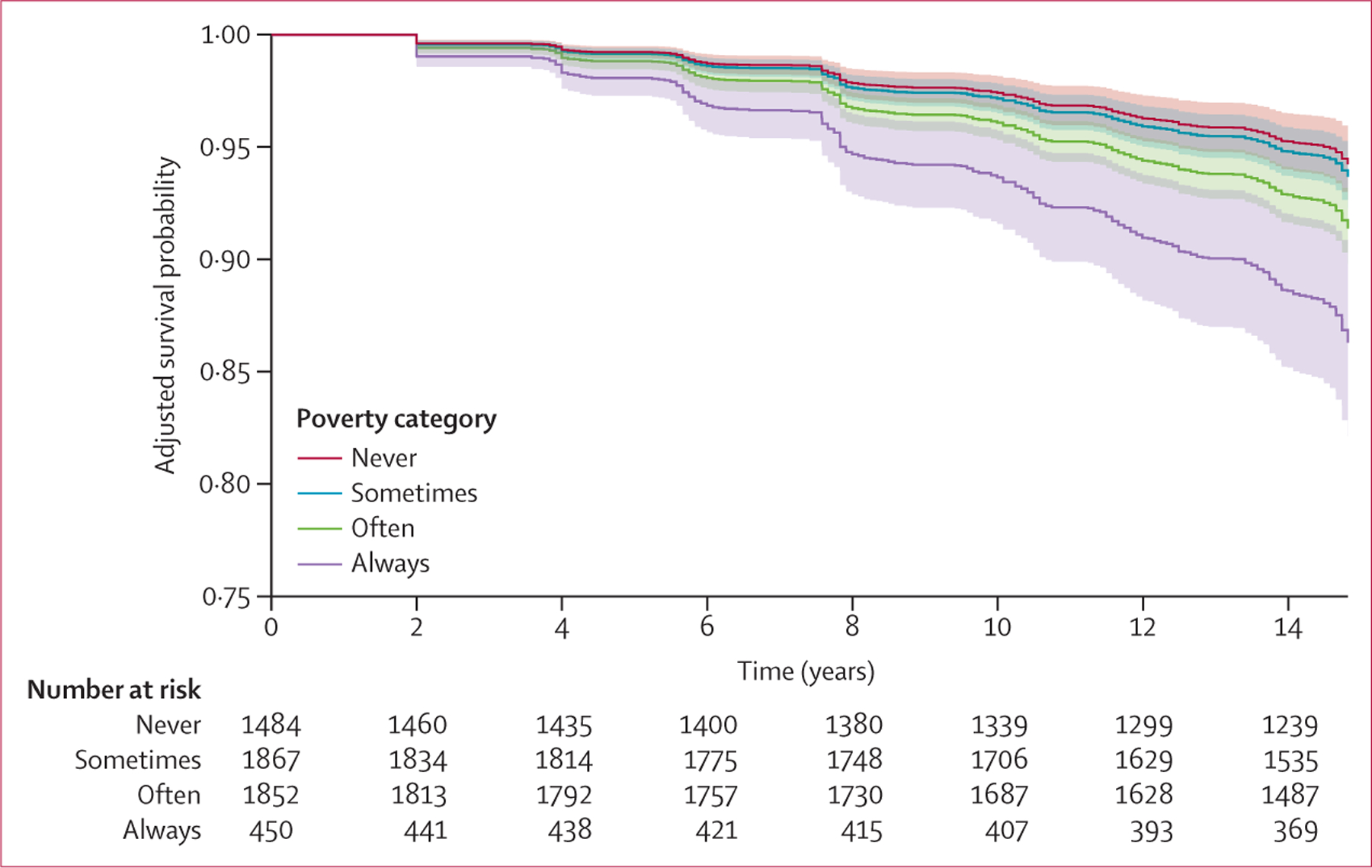
Adjusted survival probabilities for premature mortality (2004–18) by cumulative poverty category from emerging adulthood to established adulthood (1985–2004), National Longitudinal Survey of Youth, 1979 Survival probabilities adjusted for number of family income measures during exposure period, 1985 (baseline) age, race and ethnicity, sex, years of own education (measured in 1985), percentile score on the Armed Forces Qualification Test (measured in 1981), parental years of education, occupation, residence in the south of the USA at age 14 years, BMI (measured in 1985), current smoker (measured in 1984), and heavy alcohol consumption (measured in 1984). The never group had no interviews in poverty (n=1484), the sometimes group had more than zero but fewer than a third of interviews in poverty (n=1867), the often group had more than a third but fewer than all interviews in poverty (n=1852), and the always group had all interviews in poverty (n=450).

In the Cox proportional hazards regression model adjusted for participant sociodemographic characteristics, the HRs for premature mortality in those sometimes, often, and always in poverty were 1·15 (95% CI 0·83–1·59), 1·67 (1·19–2·33), and 2·90 (1·86–4·54), respectively (table 2, model 2). In the fully adjusted model, the HRs were 1·10 (95% CI 0·79–1·53) for those sometimes in poverty, 1·53 (1·09–2·14) for those often in poverty, and 2·53 (1·61–3·96) for those always in poverty (table 2, model 3). The test of the proportional hazards assumption indicated no violations for any variables included in the models. Figure 3 displays fully adjusted survival probability curves for each cumulative poverty category. Over the follow-up period, survival probabilities are lower for those often and always in poverty compared with participants never in poverty.

After imputing missing data on covariates and exposure-period family income measures, the fully adjusted HRs for premature mortality were 1·06 (95% CI 0·77–1·45) for those sometimes in poverty, 146 (1·06–2·01) for those often in poverty, and 2·16 (1·42–3·28) for those always in poverty (appendix p 8). Results when defining poverty as a family income of 175% and 150% of the Federal Poverty Level were overall similar to the main analysis (appendix pp 9–10). Associations were somewhat attenuated in the sensitivity analysis restricted to participants with 10 or more family income measures. In this analysis, the fully adjusted HR for premature poverty associated with sometimes being in poverty was 1·13 (95% CIs 0·80–1·59), for often being in poverty it was 1·57 (1·09–2·26), and for being always in poverty it was 2·25 (1·37–3·70), compared with never being in poverty (appendix p 11). Results when treating the exposure as continuous were qualitatively similar to those observed when using categorisation (results not shown). Finally, as anticipated, adjusting further for covariates measured at or near the end of the exposure period attenuated across all cumulative poverty categories (appendix p 12).

## Discussion

In this study, we found that sustained and intermittent exposure to poverty-level family income over 20 years spanning emerging adulthood through to established adulthood is associated with a higher rate of premature mortality than never being in poverty. Those with sustained exposure to poverty had a greater than two-fold higher rate of premature mortality over follow-up compared with those never in poverty. Likewise, those with a third or more interviews with poverty-level family incomes during the exposure period had a more than 1·5 times higher rate of premature mortality compared with those never in poverty. Findings were robust to adjustment for sociodemographic characteristics and were somewhat attenuated by adjustment for early-life health behaviours. Further adjustment for covariates near the end of the exposure period resulted in attenuations in estimates across cumulative poverty groups. Findings were also robust to different strategies to address missing income data over time.

Our results are broadly similar to findings from the few previous studies that have assessed cumulative poverty and low-income exposure and its association with mortality.^21,22^ Of these previous studies, our study design is most comparable to that of Brady and colleagues.^21^ Specifically, Brady and colleagues investigated the relationship between 10-year poverty exposure and mortality using data from the Panel Study of Income Dynamics. Like our study, household size-adjusted family income from multiple sources was used to define poverty, although their poverty threshold was defined as 50% of the median income rather than by the Federal Poverty Level. In their study population, which consisted of participants aged 15 years and older, being always compared with never in poverty was associated with a 1·71 times higher rate of mortality. Although their estimate for sustained poverty is lower in magnitude than what we find, their exposure period is also half of ours; thus, the larger effect estimate we find could be attributable to us capturing more of participants’ cumulative poverty exposure. In addition, differences could be due to our focus on mortality in midlife, for which evidence suggests is when differential mortality by socioeconomic status is most pronounced.^23^

Our results are also indirectly supported by previous research on cumulative poverty exposure and its association with factors that increase the risk of premature mortality. In an analysis of the NLSY79, Mossakowski observed a dose–response relationship between years in poverty during adolescence and emerging adulthood and the odds of engaging in heavy alcohol consumption as well as the frequency of heavy alcohol consumption in later emerging adulthood and early established adulthood.^24^ Heavy alcohol consumption is a major predictor of premature mortality.^25^ Other studies have found that poverty duration in younger ages also increases the risk of poor mental health and engagement in unhealthy behaviours such as smoking, both of which increase the risk for premature mortality.^26,27^ If time in poverty compounds poverty’s already negative contribution to health outcomes and behaviours,^28^ that could explain our study’s results: greater cumulative poverty exposure could result in longer and more acute exposure to conditions that increase the risk for mortality. Cumulative poverty exposure in emerging and established adulthood would then be particularly concerning as mortality from outcomes related to the behavioural risk factors associated with poverty increases after these life periods.

This study has several strengths. That our results are qualitatively consistent with existing studies is assuring; however, our study also builds upon the existing literature in important ways. Most notable is our consideration of intermittent poverty, which was aided by the length of our exposure period. Income is dynamic. Research has found that by age 65 years as many as half of US adults spend at least 1 year in poverty.^15^ Additionally, cycling in and out of poverty is not uncommon.^14^ Capturing this dynamic experience is important, and we show that experiencing intermittent poverty confers a distinct mortality risk. Another strength is our inclusion of income during emerging adulthood. Researchers increasingly recognise emerging adulthood as an important period in the life course.^29^ Emerging adults experience many life transitions, including the transition to professional employment, which represents the start of their own incomes. Economic hardship in this time impedes future earnings and carries long-term health implications.^16,17^

This study also has limitations. Although we adjusted for key potential confounders, there was likely unmeasured sources of confounding. For example, we did not have measures of income in childhood. However, we did adjust for parental education to approximate childhood socioeconomic status. Additionally, when including adjustment for covariates collected near the end of the exposure period (to roughly gauge the robustness of our findings to potential time-varying confounding), estimates were largely attenuated, and the 95% CIs included the null value. However, the included covariates are very likely part of the pathway through which earlier life poverty exposure influences mortality; as such, these attenuated estimates are likely overadjusted for mediators, which helps explain why poverty affects premature mortality rather than operates as a source of bias in our analysis. Future work should consider the use of marginal structural models or g methods to properly account for the complexities of time-varying confounders that also act as mediators, which we were unable to do in this analysis given our choice to define poverty as a categorical summary measure of increasing cumulative exposure. Due to the small sample size, we were unable to conduct analyses stratified by participant sex or race and ethnicity, two potentially important effect modifiers. In addition, due to the absence of information on causes of death in the publicly available NLSY79 data, we were unable to examine cause-specific mortality. Such information is important for informing public health interventions and should be investigated in future studies that have larger samples. We made several decisions when constructing our analytic sample that could have resulted in selection bias. Our criterion that participants contribute at least three family income measures excluded individuals hesitant to provide this information. Previous research suggests that missingness on income is not fully random and could introduce bias if associations between exposure and outcome differ among those reporting and not reporting income.^30^ For similar reasons, requiring complete data on covariates could introduce bias. Our criterion that participants remain in the cohort through to the end of the exposure period could bias estimates if those lost to follow-up differed from those who remained on factors relating to exposure and outcome. The multiple imputation analysis tested the robustness of our findings to efforts addressing the first two concerns. Reassuringly the results from the imputed data analysis were similar to the main analysis, but selection bias attributable to missing income data remains a concern because we only imputed data for participants with at least a baseline income measurement. This bias would likely operate in different directions, making it unclear how our results would be affected. Finally, although the original NLSY79 cohort was a nationally representative sample of young people from the USA, our inclusion criteria likely reduced the generalisability of our results. Notably, our requirement that participants take part in the 1985 and 2004 waves resulted in many being excluded and not recovered in our imputation analysis. Previous research has observed that individuals who are not retained in studies are more likely to come from socially and economically disadvantaged backgrounds, and that their exclusion can result in biased study outcomes.^31^

Our findings suggest that cumulative exposure to poverty across emerging adulthood and into established adulthood could increase the risk for premature mortality. Considering the large burden of poverty in the USA,^1^ determining intervenable mechanisms that underly this relationship is important.

## Supplementary Material

1

## Figures and Tables

**Table 1:** Participant characteristics, overall and by cumulative poverty category from emerging to established adulthood, National Longitudinal Survey of Youth, 1979

	Cumulative poverty category (1985–2004)				
	Overall	Never	Sometimes	Often	Always
	(N=5653)	(n=1484)	(n=1867)	(n=1852)	(n=450)
Baseline age, years	23·5 (2·2)	24·1 (2·1)	23·3 (2·2)	23·4 (2·2)	23·4 (2·2)
Sex					
Female	2987 (52·8%)	731 (49·3%)	893 (47·8%)	1029 (55·6%)	334 (74·2%)
Male	2666 (47·2%)	753 (50·7%)	974 (52·2%)	823 (44·4%)	116 (25·8%)
Race or ethnicity					
Non-Hispanic or non-Black	3084 (54·6%)	1066 (71·8%)	1182 (63·3%)	772 (41·7%)	64 (14·2%)
Hispanic	1006 (17·8%)	191 (12·9%)	300 (16·1%)	395 (21·3%)	120 (26·7%)
Black	1563 (27·6%)	227 (15·3%)	385 (20·6%)	685 (37·0%)	266 (59·1%)
Lived in the south of the USA at age 14 years	2075 (36·7%)	437 (29·4%)	648 (34·7%)	793 (42·8%)	197 (43·8%)
Years of education	12·7 (2·1)	13·7 (1·9)	13·1 (1·9)	11·8 (1·8)	10·8 (2·0)
Years of education completed by parent*	11·8 (3·4)	12·9 (3·0)	12·5 (3·2)	10·8 (3·4)	9·4 (3·3)
Armed Forces Qualification Test percentile score (measured in 1981)	44·1 (29·1)	59·7 (25·6)	51·9 (27·5)	30·8 (24·5)	15·2 (16·1)
Occupation (measured in 1985)					
Unemployed	1574 (27·8%)	150 (10·1%)	401 (21·5%)	696 (37·6%)	327 (72·7%)
Farming, production, or labour	1284 (22·7%)	334 (22·5%)	438 (23·5%)	452 (24·4%)	60 (13·3%)
Service	730 (12·9%)	135 (9·1%)	247 (13·2%)	303 (16·4%)	45 (10·0%)
Sales or clerical	1107 (19·6%)	425 (28·6%)	413 (22·1%)	254 (13·7%)	15 (3·3%)
Managerial or professional	742 (13·1%)	399 (26·9%)	262 (14·0%)	79 (4·3%)	2 (0·4%)
Other	216 (3·8%)	41 (2·8%)	106 (5·7%)	68 (3·7%)	1 (0·2%)
Current smoker† (measured in 1984)	2291 (40·5%)	460 (31·0%)	685 (36·7%)	918 (49·6%)	228 (50·7%)
Past week heavy alcohol consumption (measured in 1984)	721 (12·8%)	217 (14·6%)	262 (14·0%)	206 (11·1%)	36 (8·0%)
Participant BMI(measured in 1985), kg/m^2^	23·9 (4·3)	23·6 (3·8)	23·7 (3·9)	24·0 (4·3)	25·3 (6·0)
Number of income measures (range 3–15)	12·8 (2·4)	13·2 (2·2)	13·2 (2·1)	12·3 (2·5)	11·3 (3·0)

Data are n (%) or mean (SD). The never group had no interviews in poverty, the sometimes group had more than zero but fewer than a third of interviews in poverty, the often group had a third or more but fewer than all interviews in poverty, and the always group had all interviews in poverty.

* Education of the highest educated parent.

† Past month smoking status.

**Table 2:** Associations between cumulative poverty category from emerging to established adulthood (1985–2004) and premature mortality (2004–18) using Cox proportional hazards regression models

	Number of deaths	Model 1, HR (95% CI)	Model 2, HR (95% CI)	Model 3, HR (95% CI)
Never	64	Reference	Reference	Reference
Sometimes	91	1·13 (0·82–1·55)	1·15 (0·83–1·59)	1·10 (0·79–1·53)
Often	147	1·80 (1·34–2·42)	1·67 (1·19–2·33)	1·53 (1·09–2·14)
Always	61	3·02 (2·11–4·33)	2·90 (1·86–4·54)	2·53 (1·61–3·96)

The never group had no interviews in poverty (n=1484), the sometimes group had more than zero but fewer than a third of interviews in poverty (n=1867), the often group had a third or more but fewer than all interviews in poverty (n=1852), and the always group had all interviews in poverty (n=450). Model 1 adjusted for the number of family income measures during the exposure period. Model 2 additionally adjusted for baseline age (measured in 1985), race and ethnicity, sex, years of own education (measured in 1985), percentile score on the Armed Forces Qualification Test (measured in 1981), parental years of education, occupation, and residence in the south of the USA at age 14 years. Model 3 additionally adjusted for BMI (measured in 1985), current smoking status (measured in 1984), and heavy alcohol consumption (measured in 1984). HR=hazard ratio.

## Research in context

### Evidence before this study

We searched PubMed and Google Scholar to identify studies that examined the relationship between poverty and mortality. Our search terms included, but were not limited to, “income”, “income dynamics”, “life course”, “poverty”, “cumulative poverty”, “sustained poverty”, “earnings”, “socioeconomic status”, “socioeconomic position”, “mortality”, “premature mortality”, “emerging adulthood”, “early adulthood”, “disparities”, and “health disparities”. We did not include any date or language restrictions. Additionally, we examined the citations of the papers we found during the literature search. We conducted our search over the period between October, 2024 and May, 2025. Existing studies typically measured individuals’ incomes at single points in their life or used multiple points separated by decades (eg, childhood family socioeconomic position and a measure of family income in adulthood). Lower levels of income were associated with an increased risk for mortality. In studies that repeatedly measured income at several timepoints, maintaining consistently low income levels was associated with a greater risk for mortality than was having low income at one timepoint. Notably, no study we found focused on cumulative income from emerging adulthood (ie, those aged 18–29 years) through to established adulthood (ie, those aged 30–45 years). Socioeconomic position during emerging adulthood is increasingly recognised as an important determinant of later life economic and health outcomes, including mortality.

### Added value of this study

We build on the existing literature by using data from a national cohort study in the USA that has either annually or biannually collected detailed assessments of participants’ family income for more than 40 years starting in their adolescence and emerging adulthood in 1979 and continuing through to their midlife in 2022. In this study, we consider incomes from 1985 to 2004, when participants were on average aged 23–42 years (ie, emerging to established adulthood). Participants contributed up to 15 family income measures during that period. We then examine their risk of mortality over the next 15 years. We consider mortality during this period as premature because participants were aged between 53 years and 62 years by the end of the outcome follow-up, which is below the life expectancy for all included birth years in the cohort (1957–64). We find that those consistently in poverty across the 20-year exposure period had more than twice the risk of premature mortality compared with those never in poverty. This estimate is larger than what has been found in other studies examining the relationship between cumulative poverty and mortality. This finding could be due to the duration of our exposure and our outcome of premature mortality, which might be more influenced by socioeconomic factors than mortality at later ages. In addition, those with more intermittent exposures to poverty also had a greater risk of mortality compared with those never in poverty but a lower risk relative to those always in poverty, suggesting a potential dose-dependent mechanism.

### Implications of all the available evidence

Our findings suggest that greater cumulative exposure to poverty from emerging to established adulthood is an important determinant of health. This finding highlights the need for interventions aimed at alleviating poverty during this period in the life course. Future research should identify the behavioural and biological mediators explaining the observed association and investigate if interventions aimed directly (eg, cash transfers) or indirectly (eg, education or employment) at income result in improvements in the mediating factors.
