# Supplementary material for: Investigating associations between long-term poverty exposure and premature mortality: evidence from the National Longitudinal Survey of Youth 1979 prospective cohort
Source: Lancet Public Health. Author manuscript; Available in PMC 2025 Nov 21. (PMC12638018; doi:10.1016/S2468-2667(25)00227-0)
Supplement: 1 [file NIHMS2119847-supplement-1.pdf]

THE LANCET
Public Health

Supplementary appendix

This appendix formed part of the original submission and has been peer reviewed. We post it as supplied by the authors.

Supplement to: Colvin CL, Swift SL, Yu X, Kezios KL, Zeki Al Hazzouri A. Investigating associations between long-term poverty exposure and premature mortality: evidence from the National Longitudinal Survey of Youth 1979 prospective cohort. *Lancet Public Health* 2025; **10:** e971–78.

Supplemental Materials

Title: Investigating associations between long-term poverty exposure and premature mortality: Evidence from the National Longitudinal Survey of Youth 1979 prospective cohort

Table of Contents

Supplemental Methods……………………………………………………………………………………………2

Supplemental Table 1. Number and proportion of missingness for covariates among National Longitudinal Survey of Youth 1979 participants with at least a baseline income measure and a 2004 survey wave interview (N=6,198)…….5

Supplemental Table 2. Comparison of National Longitudinal Survey of Youth 1979 participant characteristics* among those excluded due to missing data and/or insufficient income measures and those included in the final analytic sample……………………………………………………………………………………………………6

Supplemental Table 3. Crude incidence of premature mortality (2004-2018) overall and across cumulative poverty category from emerging to established adulthood (1985-2004), National Longitudinal Survey of Youth, 1979 (N=5,653)…..7

Supplemental Table 4. Associations between cumulative poverty category from emerging to established adulthood (1985-2004) and premature mortality (2004-2018) using Cox proportion hazards regression models and multiple imputation, National Longitudinal Survey of Youth, 1979 (N = 6,198)……………………………………………….8

Supplemental Table 5. Associations between cumulative poverty category from emerging to established adulthood (1985-2004) and premature mortality (2004-2018) using Cox proportion hazards regression models and 175% of the Federal Poverty Level to define poverty, National Longitudinal Survey of Youth, 1979 (N=5,653)………………….9

Supplemental Table 6. Associations between cumulative poverty category from emerging to established adulthood (1985-2004) and premature mortality (2004-2018) using Cox proportion hazards regression models and 150% of the Federal Poverty Level to define poverty, National Longitudinal Survey of Youth, 1979 (N=5,653)………………..10

Supplemental Table 7. Associations between cumulative poverty category from emerging to established adulthood (1985-2004) and premature mortality (2004-2018) using Cox proportion hazards regression models, among participants with 10 or more family income measures out of 15, National Longitudinal Survey of Youth, 1979 (N = 5,024)………11

Supplemental Table 8. Associations between cumulative poverty category from emerging to established adulthood (1985-2004) and premature mortality (2004-2018) using Cox proportion hazards regression models, adding adjustment for covariates at the end of the exposure period, National Longitudinal Survey of Youth, 1979 (N=5,011)...…………………………………………………………………………………………….….……12

Supplemental References……………………………………………………………………………………..13

Supplemental Methods

Additional Information on Variables

i. Sex

The current analysis makes use of the NSLY79 determinations for sex. The NLSY79 determined participant sex during the study screening process. This determination was based on the perception of the study interviewer or direct respondent report if sex was "not obvious" to the interviewer. More information can be found on the NLSY79 website.[1]

ii. Race/Ethnicity

The current analysis makes use of the NLSY79 definitions for race and ethnicity. Participant race was determined during the screening process and was based on the perception of the study interviewer (either "nonblack/non-Hispanic," "black," or "other"). Ethnicity was determined by self-report. Information on how the categories ultimately chosen for the study were arrived at can be found the NLSY79 website.[2] While they are not biological determinants of morbidity and mortality, we include race/ethnicity as a covariate because of existing disparities in premature mortality by race and ethnicity that are not fully explained by socioeconomic status.[3]

iii. Early-Life Cognitive Ability (Armed Forces Qualifications Test [AFQT] score)

In line with several other studies,[4,5] we use AFQT scores as a measure of participants' early-life cognitive ability, an important predictor of morbidity and mortality as well as labor market outcomes.[6–8] AFQT scores are derived from the Armed Services Vocational Aptitude Battery (ASVAB), an exam that assesses knowledge and skills in language, science, and math for those enlisting in the US Armed Forces. The NLSY79 derived AFQT scores for participants using their scores on the verbal reasoning, arithmetic reasoning, and numeric operations sections of the test. As recommended by NLSY79 staff, we use age-adjusted AFQT scores in our analyses.[9]

iv. Occupation

For the years 1985-2000, we categorize participant occupations using the 1980 Census Occupational Codes available in the NLSY79. For 2002 and 2004 we use the 2000 Census Occupational Codes in the data.[10] Participants who reported being unemployed were labeled unemployed. Those reporting employment but whose employment did not fit a listed occupation code were labeled 'Other'. Below, we describe which categories were combined into the occupation variable we use in the current analysis.

- 1980
  - Farming, Production, Labor:
    - Farming, Forestry, and Fishing
    - Precision Production, Craft, Repair
    - Operators (All)
  - Service: Service
  - Sales/Administrative Support/Clerical:
    - Sales
    - Administrative Support/Clerical
  - Managerial and Professional Specialties/Technical
    - Managerial and Professional Specialties
    - Technical
- 2000
  - Farming, Production, Labor
    - Farming, Forestry, and Fishing
    - Construction and Extraction
    - Installation, Repair, and Maintenance
    - Production

- Transportation and Material Moving
- Food Preparation Occupations
- Setters, Operators and Tenders

- Service:
    - Healthcare Support
    - Protective Service
    - Building and Grounds Cleaning and Maintenance
    - Personal Care and Service
    - Entertainment Attendants and Related Workers
    - Funeral Related Occupations
- Sales/Administrative Support/Clerical
    - Sales and Related
    - Office and Administrative Support
- Managerial and Professional/Technical
    - Management
    - Business and Financial Operations
    - Computer and Mathematical
    - Architecture and Engineering
    - Life, Physical, and Social Services/Scientists
    - Community and Social Services
    - Legal Workers
    - Education, Training, and Library
    - Arts, Design, Entertainment, Sports, and Media
    - Healthcare Practitioners and Technical

v. Region

The NLSY79 defined region based living in the following US states:[11]

Northeast: Connecticut, Maine, Massachusetts, New Hampshire, New Jersey, New York, Pennsylvania, Rhode Island, and Vermont

North Central: Illinois, Indiana, Iowa, Kansas, Michigan, Minnesota, Missouri, Nebraska, North Dakota, Ohio, South Dakota, and Wisconsin

South: Alabama, Arkansas, Delaware, District of Columbia, Florida, Georgia, Kentucky, Louisiana, Maryland, Mississippi, North Carolina, Oklahoma, South Carolina, Tennessee, Texas, Virginia, and West Virginia

West: Alaska, Arizona, California, Colorado, Hawaii, Idaho, Montana, Nevada, New Mexico, Oregon, Utah, Washington, and Wyoming

We adjust for whether participants lived in the US South when 14 years of age because of higher morbidity and mortality as well as lower economic opportunity concentrated in the region.[12–14]

vi. Body Mass Index (BMI)

BMI was based on self-reported height and weight. While weight information was collected routinely, height was only collected periodically. Therefore, when calculating BMI, we used height from the most recent preceding survey wave when it was collected. For baseline BMI, both height and weight were collected in 1985. However, for 2004 BMI we must rely on height collected in 1985 as that was most immediate prior wave when height data were available. Some existing evidence suggests that body mass index may influence labor market outcomes.[15]

vii. Heavy Alcohol Consumption

Heavy alcohol consumption was based on sex and participant-reported amount of alcohol consumed. Those not drinking in the week preceding their interview, women who drank 7 or fewer drinks in the preceding week, and men

who drank 14 or fewer drinks in the preceding week were labeled as not having heavy alcohol consumption. Women drinking more than 7 drinks and men drinking more than 14 drinks in the week preceding their interview were labeled as heavy drinkers.[16] Associations have been observed between alcohol consumption, wages, and employment prospects.[17,18]

Supplemental Tables

Supplemental Table 1. Number and proportion of missingness for covariates among National Longitudinal Survey of Youth 1979 participants with at least a baseline income measure and a 2004 survey wave interview (N=6,198).

Covariate	Number and proportion of missingness
Baseline age, years	0 (0.0%)
Female sex	0 (0.0%)
Race/ethnicity	0 (0.0%)
Lived in South at age 14	49 (0.8%)
Years of education (1985)	11 (0.2%)
Years of education completed by parent	191 (3.1%)
AFQT percentile score	207 (3.3%)
Occupation (1985)	8 (0.1%)
Current smoker (1984)	78 (1.3%)
Heavy alcohol consumption (1984)	61 (1.0%)
Participant BMI, kg/m^2 (1985)	29 (0.5%)

AFQT: Armed Forces Qualification Test.

Supplemental Table 2. Comparison of National Longitudinal Survey of Youth 1979 participant characteristics* among those excluded due to missing data and/or insufficient income measures and those included in the final analytic sample.

**Characteristics**	**Excluded participants (N=545)**[†]	**Final analytic sample (N=5,653)**
Baseline age, years	23.8 (2.2)	23.5 (2.2)
Female sex	244 (44.8%)	2987 (52.8%)
Race/ethnicity		
Non-Hispanic/nonblack	215 (39.4%)	3,084 (54.6%)
Hispanic	130 (23.9%)	1,006 (17.8%)
Black	200 (36.7%)	1,563 (27.6%)
Lived in South at age 14	186 (37.5%)	2075 (36.7%)
Years of education (1985)	11.9 (2.4)	12.7 (2.1)
Years of education completed by parent	11.5 (3.8)	11.8 (3.4)
AFQT percentile score‡	29.4 (27.4)	44.1 (29.1)
Occupation (1985)		
Unemployed	164 (30.5%)	1,574 (27.8%)
Farming, Production, Labor	134 (25.0%)	1,284 (22.7%)
Service	78 (14.5%)	730 (12.9%)
Sales/Clerical	87 (16.2%)	1,107 (19.6%)
Managerial/Professional	31 (5.8%)	742 (13.1%)
Other	43 (8.0%)	216 (3.8%)
Current smoker (1984)	196 (42.0%)	2,291 (40.5%)
Heavy alcohol consumption (1984)	55 (11.4%)	721 (12.8%)
Participant BMI, kg/m^2 (1985)	24.2 (4.4)	23.9 (4.3)
Income measures during exposure period	11.6 (3.0)	12.8 (2.4)

AFQT: Armed Forces Qualification Test.

*We describe characteristics using Mean (SD) for numeric variables and n (%) for categorical variables.

†All cell counts do not total to 545 due to missing data.

Supplemental Table 3. Crude incidence of premature mortality (2004-2018) overall and across cumulative poverty category from emerging to established adulthood (1985-2004), National Longitudinal Survey of Youth, 1979 (N=5,653).

**Cumulative Poverty Category**	**N**	**N deaths**	**Person-years**	**Incidence Rate per 1000 person-years**	**Incident Rate Ratio**
Overall	5,653	363	76,396.42	4.75 (4.26, 5.24)	-
Never	1,484	64	20,050.83	3.19 (2.41, 3.97)	Ref
Sometimes	1,867	91	25,276.70	3.60 (2.86, 4.34)	1.13 (0.82, 1.56)
Often	1,852	147	25,007.38	5.88 (4.93, 6.83)	1.84 (1.38, 2.49)
Always	450	61	6,061.51	10.06 (7.54, 12.59)	3.15 (2.22, 4.48)

Never: no interviews in poverty; sometimes: more than zero interviews in poverty but fewer than 1/3 of interviews in poverty; often: more than 1/3 of interviews in poverty but fewer than all interviews in poverty; always: all interviews in poverty.

Supplemental Table 4. Associations between cumulative poverty category from emerging to established adulthood (1985-2004) and premature mortality (2004-2018) using Cox proportion hazards regression models and multiple imputation, National Longitudinal Survey of Youth, 1979 (N = 6,198).

	Model 1		Model 2		Model 3	
**Cumulative Poverty Category**	**HR**	**95% CI**	**HR**	**95% CI**	**HR**	**95% CI**
Never	Ref	—	Ref	—	Ref	—
Sometimes	1.10	0.81, 1.49	1.10	0.81, 1.51	1.06	0.77, 1.45
Often	1.80	1.37, 2.38	1.58	1.15, 2.17	1.46	1.06, 2.01
Always	2.96	2.13, 4.12	2.48	1.63, 3.76	2.16	1.42, 3.28

HR = Hazard Ratio, CI = Confidence Interval.
Model 1 is unadjusted.
Model 2 adjusts for 1985 (baseline) age, race/ethnicity, sex, years of own education (1985), percentile score on the Armed Forces Qualification Test (1981), parental years of education, occupation, and residence in the US south at age fourteen.
Model 3 additionally adjusts for body mass index (1985), current smoking (1984), and heavy alcohol consumption (1984).

Supplemental Table 5. Associations between cumulative poverty category from emerging to established adulthood (1985-2004) and premature mortality (2004-2018) using Cox proportion hazards regression models and 175% of the Federal Poverty Level to define poverty, National Longitudinal Survey of Youth, 1979 (N=5,653).

		Model 1		Model 2		Model 3	
**Cumulative Poverty Category**	**N Deaths**	**HR**	**95% CI**	**HR**	**95% CI**	**HR**	**95% CI**
Never	81	Ref	—	Ref	—	Ref	—
Sometimes	99	1.20	0.89, 1.61	1.22	0.90, 1.65	1.17	0.86, 1.58
Often	135	1.83	1.38, 2.42	1.68	1.22, 2.32	1.53	1.11, 2.11
Always	48	3.03	2.10, 4.38	2.92	1.85, 4.62	2.51	1.59, 3.98

HR = Hazard Ratio, CI = Confidence Interval.
Never (N=1,828): no interviews in poverty; sometimes (N=1,869): more than zero interviews in poverty but fewer than 1/3 of interviews in poverty; often (N=1,615): more than 1/3 of interviews in poverty but fewer than all interviews in poverty; always (N=341): all interviews in poverty.
Model 1 adjusts for number of family income measures during exposure period.
Model 2 adds adjustment for 1985 (baseline) age, race/ethnicity, sex, years of own education (1985), percentile score on the Armed Forces Qualification Test (1981), parental years of education, occupation, and residence in the US south at age fourteen.
Model 3 additionally adjusts for body mass index (1985), current smoking (1984), and heavy alcohol consumption (1984).

Supplemental Table 6. Associations between cumulative poverty category from emerging to established adulthood (1985-2004) and premature mortality (2004-2018) using Cox proportion hazards regression models and 150% of the Federal Poverty Level to define poverty, National Longitudinal Survey of Youth, 1979 (N=5,653).

		Model 1		Model 2		Model 3	
**Cumulative Poverty Category**	**N Deaths**	**HR**	**95% CI**	**HR**	**95% CI**	**HR**	**95% CI**
Never	98	Ref	—	Ref	—	Ref	—
Sometimes	107	1.26	0.96, 1.66	1.27	0.95, 1.69	1.21	0.91, 1.61
Often	123	1.90	1.45, 2.49	1.73	1.26, 2.37	1.54	1.12, 2.11
Always	35	2.94	1.98, 4.38	2.71	1.67, 4.38	2.45	1.51, 3.96

HR = Hazard Ratio, CI = Confidence Interval.
Never (N=2,161): no interviews in poverty; sometimes (N=1,872): more than zero interviews in poverty but fewer than 1/3 of interviews in poverty; often (N=1,373): more than 1/3 of interviews in poverty but fewer than all interviews in poverty; always (N=247): all interviews in poverty.
Model 1 adjusts for number of family income measures during exposure period.
Model 2 adds adjustment for 1985 (baseline) age, race/ethnicity, sex, years of own education (1985), percentile score on the Armed Forces Qualification Test (1981), parental years of education, occupation, and residence in the US south at age fourteen.
Model 3 additionally adjusts for body mass index (1985), current smoking (1984), and heavy alcohol consumption (1984).

Supplemental Table 7. Associations between cumulative poverty category from emerging to established adulthood (1985-2004) and premature mortality (2004-2018) using Cox proportion hazards regression models, among participants with 10 or more family income measures out of 15, National Longitudinal Survey of Youth, 1979 (N = 5,024).

		Model 1		Model 2		Model 3	
**Cumulative Poverty Category**	**N Deaths**	**HR**	**95% CI**	**HR**	**95% CI**	**HR**	**95% CI**
Never	56	Ref	—	Ref	—	Ref	—
Sometimes	84	1.15	0.82, 1.62	1.17	0.82, 1.65	1.13	0.80, 1.59
Often	127	1.89	1.37, 2.60	1.68	1.17, 2.42	1.57	1.09, 2.26
Always	47	3.08	2.07, 4.60	2.65	1.62, 4.34	2.25	1.37, 3.70

HR = Hazard Ratio, CI = Confidence Interval.
Never (N=1,364): no interviews in poverty; sometimes (N=1,737): more than zero interviews in poverty but fewer than 1/3 of interviews in poverty; often (N=1,580): more than 1/3 of interviews in poverty but fewer than all interviews in poverty; always (N=343): all interviews in poverty.
Model 1 adjusts for number of family income measures during exposure period.
Model 2 adds adjustment for 1985 (baseline) age, race/ethnicity, sex, years of own education (1985), percentile score on the Armed Forces Qualification Test (1981), parental years of education, occupation, and residence in the US south at age fourteen.
Model 3 additionally adjusts for body mass index (1985), current smoking (1984), and heavy alcohol consumption (1984).

Supplemental Table 8. Associations between cumulative poverty category from emerging to established adulthood (1985-2004) and premature mortality (2004-2018) using Cox proportion hazards regression models, adding adjustment for covariates at the end of the exposure period, National Longitudinal Survey of Youth, 1979 (N = 5,011).

		Model 1		Model 2		Model 3	
**Cumulative Poverty Category**	**N Deaths**	**HR**	**95% CI**	**HR**	**95% CI**	**HR**	**95% CI**
Never	55	Ref	—	Ref	—	Ref	—
Sometimes	76	1.10	0.78, 1.55	1.04	0.73, 1.48	0.98	0.69, 1.41
Often	126	1.89	1.37, 2.60	1.35	0.93, 1.97	1.23	0.84, 1.79
Always	47	2.92	1.96, 4.34	1.77	1.05, 2.96	1.52	0.90, 2.55

HR = Hazard Ratio, CI = Confidence Interval.

Never (N=1,344): no interviews in poverty; sometimes (N=1,688): more than zero interviews in poverty but fewer than 1/3 of interviews in poverty; often (N=1,599): more than 1/3 of interviews in poverty but fewer than all interviews in poverty; always (N=380): all interviews in poverty.

Model 1 adjusts for number of family income measures during exposure period.

Model 2 adds adjustment for 1985 (baseline) age, race/ethnicity, sex, years of own education (1985), percentile score on the Armed Forces Qualification Test (1981), parental years of education, occupation (1985), residence in the US south at age fourteen, marital status (2004) and occupation (2004).

Model 3 additionally adjusts for body mass index (1985), current smoking (1984), heavy alcohol consumption (1984), body mass index (2004), current smoking (1998), and heavy alcohol consumption (2002).

Supplemental References

1. Sex | National Longitudinal Surveys [Internet]. [cited 2025 Sep 3]. Last Accessed: 2025 Sep 4. Available from: https://www.nlsinfo.org/content/cohorts/nlsy79/topical-guide/household/sex
2. Race, Ethnicity & Immigration | National Longitudinal Surveys [Internet]. [cited 2025 Sep 3]. Last Accessed: 2025 Sep 4. Available from: https://www.nlsinfo.org/content/cohorts/nlsy79/topical-guide/household/race-ethnicity-immigration-data
3. Jemal A, Thun MJ, Ward EE, Henley SJ, Cokkinides VE, Murray TE. Mortality from Leading Causes by Education and Race in the United States, 2001. Am J Prev Med. 2008 Jan 1;34(1):1-8.e7.
4. Kremen WS, Beck A, Elman JA, Gustavson DE, Reynolds CA, Tu XM, et al. Influence of young adult cognitive ability and additional education on later-life cognition. Proc Natl Acad Sci. 2019 Feb 5;116(6):2021–6.
5. Liu L, McManus D. Impact of skills in early adulthood on lifetime homeownership disparities. J Hous Econ. 2024 Sep 1;65:102011.
6. Hemmingsson T, Melin B, Allebeck P, Lundberg I. The association between cognitive ability measured at ages 18-20 and mortality during 30 years of follow-up--a prospective observational study among Swedish males born 1949-51. Int J Epidemiol. 2006 Jun;35(3):665–70.
7. Hemmingsson T, Melin B, Allebeck P, Lundberg I. Cognitive ability in adolescence and mortality in middle age: a prospective life course study. J Epidemiol Community Health. 2009 Sep 1;63(9):697–702.
8. Lin D, Lutter R, Ruhm CJ. Cognitive performance and labour market outcomes. Labour Econ. 2018 Apr 1;51:121–35.
9. Aptitude, Achievement & Intelligence Scores: National Longitudinal Survey of Youth 1979 [Internet]. [cited 2024 Jul 2]. Last Accessed: 2025 Sep 4. Available from: https://www.nlsinfo.org/content/cohorts/nlsy79/topical-guide/education/aptitude-achievement-intelligence-scores
10. Occupations | National Longitudinal Surveys [Internet]. [cited 2025 Sep 3]. Last Accessed: 2025 Sep 4. Available from: https://www.nlsinfo.org/content/cohorts/nlsy79/topical-guide/employment/occupations
11. NLSY79 Attachment 100: Geographic Regions | National Longitudinal Surveys [Internet]. [cited 2025 Sep 3]. Last Accessed: 2025 Sep 4. Available from: https://www.nlsinfo.org/content/cohorts/nlsy79/other-documentation/codebook-supplement/nlsy79-attachment-100-geographic
12. Fenelon A. Geographic Divergence in Mortality in the United States. Popul Dev Rev. 2013 Dec 1;39(4):611–34.
13. Connor DS, Storper M. The changing geography of social mobility in the United States. Proc Natl Acad Sci U S A. 2020 Dec 1;117(48):30309–17.
14. Farina MP, Crimmins EM, Hayward MD. The role of Southern context in shaping life course exposures linked to dementia incidence for Black and White older adults in the United States. BMC Geriatr. 2024 Nov 11;24(1):927.
15. Campbell DD, Green M, Davies N, Demou E, Ward J, Howe LD, et al. Effects of increased body mass index on employment status: a Mendelian randomisation study. Int J Obes 2005. 2021;45(8):1790–801.
16. CDC. Alcohol Use and Your Health [Internet]. Alcohol Use. 2025 [cited 2025 Sep 3]. Last Accessed: 2025 Sep 4. Available from: https://www.cdc.gov/alcohol/about-alcohol-use/index.html
17. Jørgensen MB, Pedersen J, Thygesen LC, Lau CJ, Christensen AI, Becker U, et al. Alcohol consumption and labour market participation: a prospective cohort study of transitions between work, unemployment, sickness absence, and social benefits. Eur J Epidemiol. 2019;34(4):397–407.

18. Campbell DD, Green M, Davies NM, Harrison S, Demou E, Howe LD, et al. Effects of alcohol consumption on employment and social outcomes: a Mendelian randomisation study. Alcohol Alcohol. 2025 Sep 1;60(5):agaf038.
